# Risk of Diabetes Mellitus in the Myasthenia Gravis: A Systematic Review and Meta-Analysis

**DOI:** 10.3390/jcm14124221

**Published:** 2025-06-13

**Authors:** Vasileios Giannopapas, Maria-Ioanna Stefanou, Vasiliki Zouvelou, Georgia Papagiannopoulou, Vassiliki Smyrni, Maria Kosmidou, Dimitrios K. Kitsos, Anna Keramida, Stavroula Salakou, Georgios Tsivgoulis, John S. Tzartos, Sotirios Giannopoulos

**Affiliations:** 1 Second Department of Neurology, Attikon University Hospital, National and Kapodistrian University of Athens, 10679 Athens, Greece; bgiannopapas@gmail.com (V.G.); marianna421@hotmail.co.uk (M.-I.S.); georgiapap22@hotmail.com (G.P.); b.smyrni@hotmail.com (V.S.); dkitsos@icloud.com (D.K.K.); akeramida@outlook.com (A.K.); ssalakou@gmail.com (S.S.); tsivgoulisgiorg@yahoo.gr (G.T.); jtzartos@gmail.com (J.S.T.); 2 Department of Neurology & Stroke, Eberhard-Karls University of Tübingen, 72074 Tübingen, Germany; 3 Hertie Institute for Clinical Brain Research, Eberhard-Karls University of Tübingen, 72074 Tübingen, Germany; 4 First Department of Neurology, Eginition Hospital, National and Kapodistrian University of Athens, 10679 Athens, Greece; vzouvelu@med.uoa.gr; 5 Third Department of Internal Medicine, Aristotle University of Thessaloniki, 54124 Thessaloniki, Greece; mskosmidou@gmail.com

**Keywords:** myasthenia gravis, diabetes mellitus, autoimmune disorders

## Abstract

**Background/Objectives:** Myasthenia gravis (MG) comprises an autoimmune disorder marked by muscle weakness and fatigue. MG frequently coexists with other autoimmune conditions, such as diabetes mellitus, which may exacerbate the clinical burden of MG and adversely impact overall quality of life. **Methods**: This systematic review and meta-analysis aimed to assess the prevalence of diabetes in patients with MG. Following PRISMA guidelines, a comprehensive search was conducted in the MEDLINE PubMed, Scopus, and Google Scholar databases. **Results**: A total of 11 studies comprising 16,825 patients were included. The pooled prevalence of diabetes among MG patients was 15.6% (95% CI [8.4%, 24.7%]; I^2^ = 99%, *p* < 0.001). Compared to healthy controls, MG patients had a 1.24-fold increased risk of developing diabetes (95% CI [1.01–1.54], *p* < 0.001). Meta-regression showed no association between age and diabetes prevalence, although potential publication bias was noted (Egger’s test, *p* = 0.72). **Conclusions**: This meta-analysis reveals a higher prevalence of diabetes among MG patients, with an elevated risk compared to healthy individuals. These findings suggest that the association between MG and diabetes may be mediated by corticosteroid use, genetic factors, and reduced physical activity. The main limitations of this study are the lack of reported data regarding the type of diabetes and patients’ demographic and disease characteristics (MG classification severity and duration and type of treatment). Further studies are needed to investigate the underlying causal mechanisms and to elucidate the relationship between MG and diabetes subtypes.

## 1. Introduction

Myasthenia gravis (MG) is a rare autoimmune neuromuscular disorder characterized by fatigable weakness of voluntary muscles, primarily due to autoantibodies either to acetylcholine receptors (AChR Abs), muscle-specific kinase antibodies (MuSK-Abs), low density lipoprotein receptor-related protein 4 (LRP-4-Abs), or titin antibodies [1,2,3,4]. Myasthenia gravis (MG) can be classified based on clinical presentation into ocular MG, which is limited to the extraocular muscles, and generalized MG, which involves additional muscle groups including bulbar, neck, limb, and respiratory muscles [1,4]. In addition to differences in clinical manifestations, treatment approaches also vary, with ocular MG typically requiring lower doses of corticosteroids compared to the generalized form [1,4]. Comorbid disorders, such as diabetes mellitus, are frequently encountered in MG, especially in late and late-onset MG patients (onset of disease > 50 and ≥65 years, respectively). The existence of such comorbid conditions can increase morbidity, mortality, and influence therapeutic decisions, negatively impacting patients’ daily functioning and overall quality of life [5].

Although research on the prevalence and potential associations between MG and various comorbidities, including diabetes mellitus, remains limited, there has been a growing shift in research interest toward this topic in recent years [1]. Diabetes mellitus, in particular, is a chronic metabolic disorder characterized by hyperglycemia, which has been reported to occur frequently in patients with MG. While there is a possibility that the connection between MG and diabetes is mediated by a pathophysiological predisposition shared between autoimmune conditions, the underlying etiology behind this increased incidence is likely more complex, entailing genetic factors, effects of treatments commonly used in MG (such as corticosteroids), and lifestyle factors [6,7,8].

Despite the growing body of literature on this topic, previous studies have reported varying rates of diabetes incidence within the myasthenia gravis (MG) population. However, these findings have not been systematically analyzed, resulting in uncertainty regarding the true prevalence of diabetes among MG patients. To address this issue, the present study will conduct a systematic review and meta-analysis to estimate the prevalence of diabetes in this population. This systematic review and meta-analysis aim to provide valuable insights into the frequency of diabetes and its relationship with MG, thereby informing current clinical practices and supporting personalized treatment approaches.

## 2. Materials and Methods

### 2.1. Standard Protocol Approvals and Registrations

The pre-specified protocol of this systematic review and meta-analysis is registered in the Open Search Foundation (OSF) (registration: OSF.IO/NYZRG). This meta-analysis was reported according to the updated Preferred Reporting Items for Systematic Reviews and Meta-Analyses (PRISMA) guidelines [9] and was written following the Meta-analysis of Observational Studies in Epidemiology (MOOSE) proposal [10]. This study did not require an ethical board approval or written informed consent.

### 2.2. Data Sources, Searches, and Study Selection

A systematic literature search was conducted to identify eligible studies reporting on diabetes in patients with MG. The literature search was performed by two independent reviewers (VG, VS) and included three databases: the MEDLINE PubMed, Scopus, and Google Scholar (first 200 results). The search included the following terms: “myasthenia gravis”, “generalized myasthenia gravis”, and “diabetes mellitus”. The complete search algorithm is presented in the Supplementary Materials. No language or other restrictions were applied. The search spanned from the inception of each database to 1 September 2024. Furthermore, the reference lists of published articles were manually searched. Clinical trials, population-based studies or registries, and observational cohort studies reporting on diabetes in patients with MG versus healthy controls were eligible for inclusion.

Based on the predefined protocol, studies were excluded if they (1) did not include patients with a definite MG diagnosis; (2) reported outcomes not aligned with our inclusion criteria; (3) were case series, case reports, commentaries, narrative and systematic reviews, non-peer reviewed studies, pre-prints, and conference abstracts; (4) had purposive sampling; and (5) had a sample size smaller than 30 patients. In the case of overlapping data between studies, the study with the largest dataset was retained. Retrieved records were assessed by the two independent reviewers (VG, VS), and any disagreements were resolved by the corresponding author (SG).

### 2.3. Quality Control, Bias Assessment, and Data Extraction

The risk of bias for the relevant domains of each included study was assessed by two independent reviewers (VG, VS) using the Risk of Bias In Non-randomized Studies of Interventions (ROBINS-I) tool [11]. Disagreements were settled by the corresponding author (SG). Data from individual studies, including author names, date of publication, study design, country, events (diabetes cases, type of diabetes), and patient characteristics were extracted in structured reports.

### 2.4. Outcomes

An aggregate data meta-analysis was performed with the inclusion of the identified population-based studies or registries and observational cohort studies.

The predefined primary outcome measures were (i) the pooled prevalence of diabetes in the MG patient population and (ii) the relative risk of diabetes in patients with MG versus healthy controls. Secondary outcomes included potential relationships between the risk of diabetes and demographic characteristics.

### 2.5. Statistical Analysis

For the aggregate meta-analysis, the pooled prevalence with the corresponding 95% confidence interval (95% CI) was calculated using the Freeman–Tukey variance-stabilizing double arcsine transformation [12,13]. The random-effects model of meta-analysis (DerSimonian and Laird) was used to calculate the pooled estimates [14]. Pairwise comparisons were conducted between MG patients and the general population and were reported using risk ratios (RRs) and corresponding 95% confidence intervals (95% CIs). Heterogeneity was assessed with the I^2^ index (values > 50% and values > 75% were considered to represent substantial and considerable heterogeneity, respectively) and Cochran Q statistics. The significance level for the Q statistic was set at 0.1. Publication bias across individual studies was assessed by Funnel plot inspection and the result of the Egger’s linear regression test [15]. All statistical analyses and figure production were carried out using RStudio for IOS [R studio/R Meta package] (v.4.5.0) [13,15].

### 2.6. Data Availability Statement

All data generated or analyzed during this study are included in the article and its Supplementary Materials files.

## 3. Results

**Figure 1:**
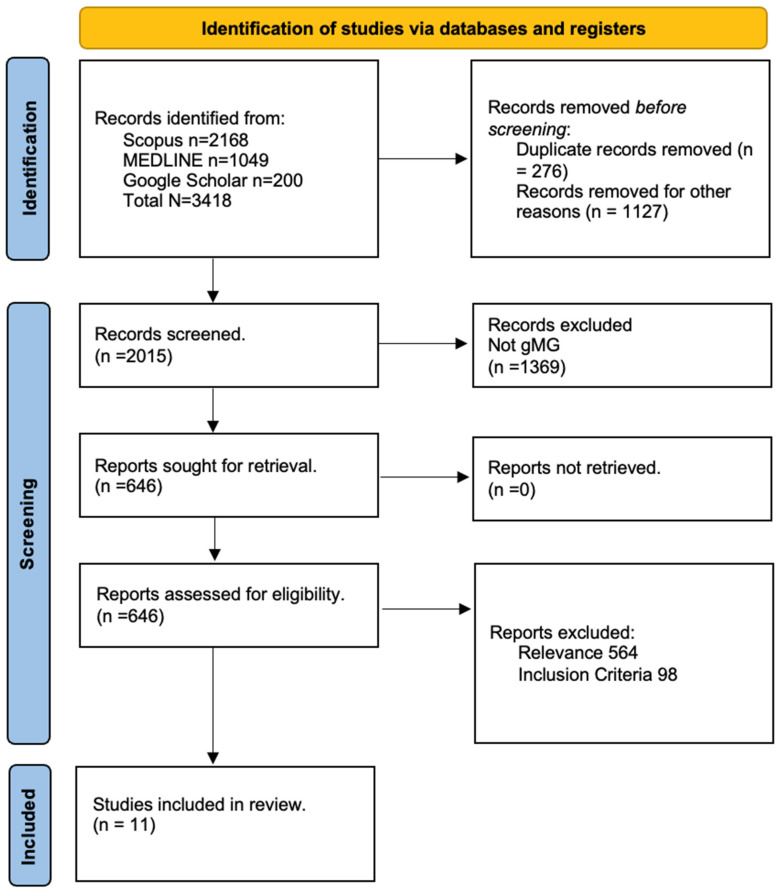
Prisma flow-chart.

A total of 3418 records were identified by the systematic literature search. After duplicate removal and the implementation of the inclusion–exclusion criteria, 36 articles were further assessed. Finally, 11 studies were included in the meta-analysis (Figure 1, Table 1).

### 3.1. Quality Assessment

Studies were assessed using the Robins-I tool and presented with a low-to-moderate risk of bias due to the confounding and reporting of outcomes (Supplementary Figure S1, Supplementary Figure S2).

### 3.2. Quantitative Results

#### Primary Outcomes

A total of 11 studies [1,16,17,18,19,20,21,22,23,24,25], including 16,825 patients diagnosed with MG and 291,626 controls, were included (Table 1). The majority of the studies did not differentiate between diabetes mellitus type I and type II. Studies did not report stratified data between diabetes occurrence and MG type (ocular versus generalized), MG severity, MG treatment (acetylcholinesterase inhibitors, steroid treatment, and non-steroid immunosuppressive treatment), or the duration of treatment. In four studies that reported the type of MG [17,18,21,25] treatment, the percentage of participants receiving non-steroid immunosuppressive therapy ranged from 3% to 51%.

**Figure 2:**
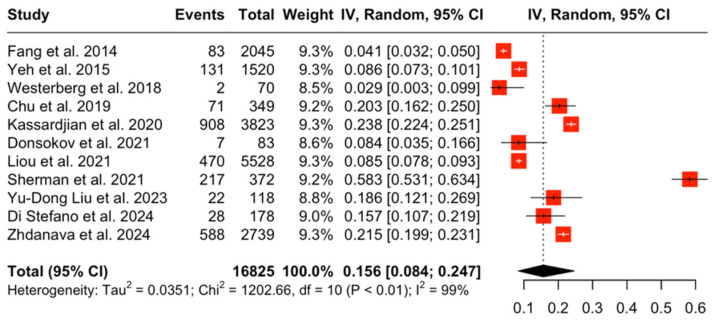
Pooled prevalence of diabetes in patients with MG [1,16,17,18,19,20,21,22,23,24,25].

**Figure 3:**
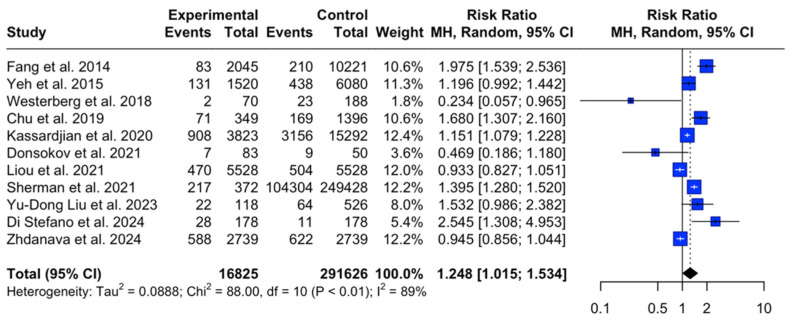
Relevant risk of diabetes in patients with MG versus healthy controls [1,16,17,18,19,20,21,22,23,24,25].

The pooled prevalence of diabetes (regardless of type) among patients with MG was 15.6% (95% CI [8.4%, 24.7%], I^2^ = 99%, *p* < 0.001) (Figure 2), while in the control group, the aggregated pooled prevalence was 13.5% (95% CI [7.9%, 20.3%]; I^2^ = 100%, *p* < 0.001). The relative risk of diabetes in patients with MG versus controls was found to be 1.24 (95% CI [1.01, 1.54]; I^2^ = 89% *p* < 0.001), which indicates that patients with MG have a 1.24-fold increased risk of developing diabetes compared to healthy individuals (Figure 3).

### 3.3. Secondary Outcomes

In a subset of six [17,18,20,21,24,25] studies that provided data regarding the patients’ age, the potential association between diabetes risk and age was assessed using meta-regression with no statistically significant associations detected (β = 0.004, *p* = 0.12).

### 3.4. Publication Bias Assessment

Funnel plot inspection and Egger’s linear regression test (β = 0.55, *p* = 0.72) were both indicative of low-degree risk of publication bias for the relative risk of diabetes in patients with MG.

## 4. Discussion

To our knowledge, this is the first systematic review and meta-analysis to examine both the prevalence and relative risk of diabetes in patients with MG. We analyzed data from 11 studies, comprising 16,825 patients with MG, and demonstrated that the pooled prevalence of diabetes in this population was 15.6%. This estimate significantly exceeds the global prevalence of diabetes mellitus, which was estimated by the 2021 Global Burden of Disease Collaborative Network study and ranges between 9% and 10%, as well as the prevalence of diabetes in high-income countries, which amounts to 10.4% [26]. Furthermore, our findings indicate that patients with MG have a 1.24-fold increased risk of developing diabetes compared to healthy controls. These findings have significant clinical relevance as they provide robust evidence for the co-occurrence of diabetes and MG, as ascertained by the large sample size (16,825 MG patients and 291,626 healthy controls) and the low degree of publication bias in the included studies. However, it is important to note that the studies included in this systematic review and meta-analysis reported diabetes as part of the baseline characteristics assessment, i.e., without consistently specifying the type of diabetes or indicating whether the condition predated study participation. This limitation restricts our ability to draw firm conclusions about the causality or directionality of the association between MG and diabetes.

When interpreting the aforementioned evidence, it is crucial to consider the potential bidirectional relationship between MG and diabetes. Numerous studies have demonstrated that individuals frequently develop diabetes before a diagnosis of MG, resulting in heightened susceptibility to MG compared to those without diabetes [1]. Conversely, other research suggests that individuals with MG are at an increased risk of developing diabetes during the course of MG [17]. Several hypotheses have been proposed in the literature regarding the underlying associations between MG and both type I and type II diabetes. Specifically, genetic associations have been suggested for type I diabetes and MG, while type II diabetes may be linked to the cardinal clinical features of MG and treatment-related adverse events.

Regarding genetic susceptibility, previous studies have suggested a shared genetic predisposition between MG and type I diabetes. Wang et al. (2022) [27] and Chuang Wen Yu et al. [28] identified a link between the +49 A/G coding polymorphism in the CTLA4 gene and thymoma-positive MG, as well as other autoimmune conditions, including type I diabetes. This polymorphism occurs in the first exon, resulting in an amino acid change (Thr17Ala) that alters the glycosylation pattern of the mature protein, reducing its expression on the cell membrane [29,30]. Furthermore, studies indicate that the PTPN22 T allele polymorphism is associated with non-thymoma MG in the absence of anti-titin antibodies [31]. This missense polymorphism changes arginine (R) to tryptophan (W) at position 620, impairing its interaction with the tyrosine kinase Csk, which enhances PTPN22 activation and decreases interleukin-2 production, thus increasing susceptibility to type I diabetes [32].

On the other hand, the relationship between type II diabetes mellitus and MG appears to be more closely linked to corticosteroid use and reduced physical activity stemming from fatigable skeletal muscle weakness [17]. Corticosteroids are the first-line immunosuppressive therapy for MG due to their rapid clinical effect and high efficacy. However, chronic use of corticosteroids is associated with numerous severe side effects, including elevated serum glucose levels [6]. Previous studies have indicated that corticosteroid use can double the risk of developing type II diabetes, with risk ratio estimates ranging from 1.36 to 2.31. A more recent meta-analysis revealed that 32.3% of patients treated with glucocorticoids developed hyperglycemia, while 18.6% developed glucocorticoid-induced type II diabetes [6]. Furthermore, another study, which compared MG patients receiving corticosteroid treatment to those who did not, demonstrated that corticosteroid use significantly increased the risk of diabetes occurrence in these patients (HR = 1.46; 95% CI = 1.15–1.86) [17].

Another potential pathophysiological link between MG and type II diabetes mellitus involves advanced glycation end products (AGEs), which are markedly elevated in the early stages of type II diabetes and are hypothesized to contribute to MG pathogenesis [1]. AGEs serve as ligands for the receptor for AGEs (RAGE), triggering inflammatory signaling pathways that lead to the production of cytokines and growth factors. Furthermore, AGEs stimulate the proliferation of CD4(+)CD28(-) T cells, which play a role in autoimmune diseases [5,19]. Bouchikh and colleagues [33] reported that MG patients with elevated RAGE expression in thymic tissue also showed increased levels of AChR antibodies, suggesting that AGEs-RAGE signaling may be crucial in MG pathophysiology. However, further studies are needed to clarify this relationship and to explore the role of AGEs in immune system activation and their potential impact on the MG-diabetes association.

Finally, in addition to treatment effects, the reduced physical activity levels and increased fatigue experienced by patients with myasthenia gravis (MG) may also increase the risk of type II diabetes mellitus [7,34,35]. Exercise capacity in MG patients can be limited due to fatigability and the involvement of limb and respiratory muscles, leading to further reductions in physical activity. This inactivity can exacerbate corticosteroid-induced hyperglycemia, bone loss, and muscle atrophy. Notably, Li et al. demonstrated that even MG patients not receiving corticosteroid treatment exhibited a higher prevalence of glucose and lipid metabolic disorders, likely due to insulin resistance linked to physical inactivity [36]. Therefore, MG patients with sustained clinical improvement should be encouraged to engage in physical activity through individualized training programs [37]. Given that a sedentary lifestyle combined with reduced physical activity has been associated with poor glycemic control, metabolic imbalance, and an increased risk of type II diabetes mellitus, these factors may contribute to the observed associations between MG and diabetes [8].

### Limitations

Some limitations of the present systematic review and meta-analysis should be acknowledged. First, there was a substantial degree of heterogeneity among the included studies. Second, most studies did not differentiate between type I and type II diabetes mellitus. Third, as indicated by the risk of bias analysis, several studies failed to report key data on patients’ demographic and disease characteristics (e.g., MG classification, MG severity, duration of treatment, and type of treatment (acetylcholinesterase inhibitors, steroid treatment, non-steroid immunosuppressive treatment)) in relation to diabetes prevalence. Finally, although we found no association between diabetes risk and age, this conclusion was based on a small subset of studies that provided data for meta-regression. Therefore, future larger, well-designed controlled studies are warranted to corroborate these findings.

## 5. Conclusions

This study highlights the significant prevalence of diabetes among patients with MG, emphasizing a concerning association that warrants further investigation. The elevated risk of diabetes in this cohort may be linked to several factors, including (a) underlying genetic predispositions, (b) extensive corticosteroid therapy, and (c) decreased physical activity due to MG. However, the current analysis cannot establish causality, as data on the temporal relationship between the onset of diabetes and MG are lacking. Future research should aim to systematically explore the sequence of disease onset, the type of diabetes, and pertinent clinical factors—such as cumulative corticosteroid exposure, non-steroid immunosuppressive treatment and treatment duration—in patients with MG and concurrent diabetes. This comprehensive approach will enhance our understanding of the interplay between these conditions and guide the development of targeted preventive and management strategies.

## Figures and Tables

**Table 1:** Study characteristics [1,16,17,18,19,20,21,22,23,24,25].

Author	MG Sample	Diabetes Cases in MG	MG Sample Age (Years)	Control Group Sample	Diabetes Cases in Control Group
Fang et al. 2014	2045	83		10,221	210
Yeh et al. 2015	1520	131	45.7	6080	438
Westerberg et al. 2018	70	2	47.5	188	23
Chu et al. 2019	349	71		1396	169
Kassardjian et al. 2020	3823	908	63.8	15,292	3156
Donsokov et al. 2021	83	7	58	50	9
Liou et al. 2021	5528	470		5528	504
Sherman et al. 2021	372	217		249,428	104,304
Yu-Dong Liu et al. 2023	118	22		526	64
Di Stefano et al. 2024	178	28	59.2	178	11
Zhdanava et al. 2024	2739	588	56.2	2739	622

## Data Availability

All data generated or analyzed during this study are included in this article and its Supplementary Information files.

## Supplementary Materials

The following supporting information (search algorithm, risk of bias plot and funnel plot) can be downloaded at https://www.mdpi.com/article/10.3390/jcm14124221/s1; Figure S1: Risk of bias traffic plot.; Figure S2: Risk of bias summary plot.; Figure S3: Funnel plot.

## Abbreviations

The following abbreviations are used in this manuscript:

MDPI	Multidisciplinary Digital Publishing Institute
DOAJ	Directory of open access journals
TLA	Three letter acronym
LD	Linear dichroism

## References

[1] Liu Y.-D., Tang F., Li X.-L., Liu Y.-F., Zhang P., Yang C.-L., Du T., Li H., Wang C.-C., Liu Y. (2023). Type 2 diabetes mellitus as a possible risk factor for myasthenia gravis: A case–control study. Front. Neurol..

[2] Dresser L., Wlodarski R., Rezania K., Soliven B. (2021). Myasthenia Gravis: Epidemiology, Pathophysiology and Clinical Manifestations. J. Clin. Med..

[3] Alkhotani A.M., Alrishi N. (2023). Severity and antibodies profile of seropositive myasthenia gravis. Saudi J. Health Sci..

[4] Toth C., McDonald D., Oger J., Brownell K. (2006). Acetylcholine receptor antibodies in myasthenia gravis are associated with greater risk of diabetes and thyroid disease. Acta Neurol. Scand..

[5] Misra U.K., Kalita J., Singh V.K., Kumar S. (2020). A study of comorbidities in myasthenia gravis. Acta Neurol. Belg..

[6] Barker H.L., Morrison D., Llano A., Sainsbury C.A.R., Jones G.C. (2023). Practical Guide to Glucocorticoid Induced Hyperglycaemia and Diabetes. Diabetes Ther..

[7] Andersen L.K., Aadahl M., Vissing J. (2021). Fatigue, physical activity and associated factors in 779 patients with myasthenia gravis. Neuromuscul. Disord..

[8] Sharif S., Sharif H., Rehman J., Fatima Z. (2023). Is a sedentary lifestyle a leading causal factor of obesity and distress in type 2 diabetes? A cross-sectional study in low-socioeconomic areas of Karachi, Pakistan. BMJ Public Health.

[9] Giannopapas V., Stefanou M., Smyrni V., Kitsos D.K., Kosmidou M., Stasi S., Chasiotis A.K., Stavrogianni K., Papagiannopoulou G., Tzartos J.S. (2024). Waist Circumference and Body Mass Index as Predictors of Disability Progression in Multiple Sclerosis: A Systematic Review and Meta-Analysis. J. Clin. Med..

[10] Giannopapas V., Palaiodimou L., Kitsos D., Papagiannopoulou G., Stavrogianni K., Chasiotis A., Kosmidou M., Tzartos J.S., Paraskevas G.P., Bakalidou D. (2023). The Prevalence of Diabetes Mellitus Type II (DMII) in the Multiple Sclerosis Population: A Systematic Review and Meta-Analysis. J. Clin. Med..

[11] Sterne J.A., Hernán M.A., Reeves B.C., Savović J., Berkman N.D., Viswanathan M., Henry D., Altman D.G., Ansari M.T., Boutron I. (2016). ROBINS-I: A tool for assessing risk of bias in non-randomised studies of interventions. BMJ.

[12] Freeman M.F., Tukey J.W. (1950). Transformations related to the angular and the square root. Ann. Math. Stat..

[13] Borenstein M., Higgins J.P. (2013). Meta-analysis and subgroups. Prev. Sci..

[14] Tsivgoulis G., Katsanos A.H., Köhrmann M., Caso V., Perren F., Palaiodimou L., Deftereos S., Giannopoulos S., Ellul J., Krogias C. (2019). Duration of Implantable Cardiac Monitoring and Detection of Atrial Fibrillation in Ischemic Stroke Patients: A Systematic Review and Meta-Analysis. J. Stroke.

[15] Egger M., Smith G.D., Schneider M., Minder C. (1997). Bias in meta-analysis detected by a simple, graphical test. BMJ.

[16] Fang F., Sveinsson O., Thormar G., Granqvist M., Askling J., Lundberg I.E., Ye W., Hammarström L., Pirskanen R., Piehl F. (2015). The autoimmune spectrum of myasthenia gravis: A Swedish population-based study. J. Intern. Med..

[17] Yeh J., Chen H., Lin C., Chen Y., Chiu H., Kao C. (2015). Risk of diabetes mellitus among patients with myasthenia gravis. Acta Neurol. Scand..

[18] Westerberg E., Landtblom A., Punga A.R. (2018). Lifestyle factors and disease-specific differences in subgroups of Swedish Myasthenia Gravis. Acta Neurol. Scand..

[19] Chu H., Tseng C., Liang C., Yeh T., Hu L., Yang A.C., Tsai S.-J., Shen C.-C. (2019). Risk of Depressive Disorders Following Myasthenia Gravis: A Nationwide Population-Based Retrospective Cohort Study. Front. Psychiatry.

[20] Kassardjian C.D., Widdifield J., Paterson J.M., Kopp A., Nagamuthu C., Barnett C., Tu K., Breiner A. (2020). Serious infections in patients with myasthenia gravis: Population-based cohort study. Eur. J. Neurol..

[21] Donskov A.O., Vinge L., Axelsen S.M., Andersen H. (2021). Overactive bladder in patients with myasthenia gravis—A cross-sectional population-based study. Acta Neurol. Scand..

[22] Liou Y., Wei J.C., Hu K., Hung Y., Chou M., Chang R. (2021). Risk of subsequent atrial fibrillation in patients with myasthenia gravis. Medicine.

[23] Sherman W.F., Wu V.J., Ofa S.A., Ross B.J., Savage-Elliott I.D., Sanchez F.L. (2021). Increased rate of complications in myasthenia gravis patients following hip and knee arthroplasty: A nationwide database study in the PearlDiver Database on 257,707 patients. Acta Orthop..

[24] Di Stefano V., Iacono S., Militello M., Leone O., Rispoli M.G., Ferri L., Ajdinaj P., Lanza P., Lupica A., Crescimanno G. (2024). Comorbidity in myasthenia gravis: Multicentric, hospital-based, and controlled study of 178 Italian patients. Neurol. Sci..

[25] Zhdanava M., Pesa J., Boonmak P., Cai Q., Pilon D., Choudhry Z., Souayah N. (2024). Economic burden of generalized myasthenia gravis (MG) in the United States and the impact of common comorbidities and acute MG-events. Curr. Med. Res. Opin..

[26] Institute for Health Metrics and Evaluation (2024). Global Burden of Disease Collaborative Network Global Burden of Disease Study 2021. Results. https://vizhub.healthdata.org/gbd-results/.

[27] Wang X., Kakoulidou M., Qiu Q., Giscombe R., Huang D., Pirskanen R., Lefvert A.K. (2002). CDS1 and promoter single nucleotide polymorphisms of the CTLA-4 gene in human myasthenia gravis. Genes Immun..

[28] Chuang W., Ströbel P., Gold R., Nix W., Schalke B., Kiefer R., Opitz A., Klinker E., Müller-Hermelink H.K., Marx A. (2005). A CTLA4^high^ genotype is associated with myasthenia gravis in thymoma patients. Ann. Neurol..

[29] Kouki T., Sawai Y., Gardine C.A., Fisfalen M.-E., Alegre M.-L., DeGroot L.J. (2000). CTLA-4 gene polymorphism at position 49 in exon 1 reduces the inhibitory function of CTLA-4 and contributes to the pathogenesis of Graves’ disease. J. Immunol..

[30] Anjos S., Nguyen A., Ounissi-Benkalha H., Tessier M., Polychronakos C. (2002). A Common Autoimmunity Predisposing Signal Peptide Variant of the Cytotoxic T-lymphocyte Antigen 4 Results in Inefficient Glycosylation of the Susceptibility Allele. J. Biol. Chem..

[31] Vandiedonck C., Capdevielle C., Giraud M., Krumeich S., Jais J., Eymard B., Tranchant C., Gajdos P., Garchon H. (2006). Association of the PTPN22*R620W polymorphism with autoimmune myasthenia gravis. Ann. Neurol..

[32] Bottini N., Musumeci L., Alonso A., Rahmouni S., Nika K., Rostamkhani M., MacMurray J., Meloni G.F., Lucarelli P., Pellecchia M. (2004). A functional variant of lymphoid tyrosine phosphatase is associated with type I diabetes. Nat. Genet..

[33] Bouchikh M., Zouaidia F., Benhaddou E.H.A., Mahassini N., Achir A., El Malki H.O. (2017). Expression of receptor for advanced glycation end-products (RAGE) in thymus from myasthenia patients. Rev. Neurol..

[34] Alsop T., Williams K., Gomersall S. (2022). Physical Activity and Sedentary Behaviour in People with Myasthenia Gravis: A Cross-Sectional Study. J. Neuromuscul. Dis..

[35] Alekseeva T.M., Gavrilov Y.V., Kreis O.A., Valko P.O., Weber K.P., Valko Y. (2018). Fatigue in patients with myasthenia gravis. J. Neurol..

[36] Li L.J., Guan Y.Z., Lü F., Song Y.W., Xu X.J., Jiang Y., Wang O., Xia W.B., Xing X.P., Li M. (2018). Glucose and lipid metabolic disorders in myasthenia gravis patients and its mechanisms. Zhonghua yi xue za zhi.

[37] O’Connor L., Westerberg E., Punga A.R. (2020). Myasthenia Gravis and Physical Exercise: A Novel Paradigm. Front. Neurol..

