# Supplementary material for: Risk of Diabetes Mellitus in the Myasthenia Gravis: A Systematic Review and Meta-Analysis
Source: J Clin Med. 2025 Jun 13;14(12):4221. doi: 10.3390/jcm14124221 (PMC12194735; doi:10.3390/jcm14124221)
Supplement: Supplementary file 1 [file jcm-14-04221-s001.zip › jcm-3649219-supplementary.pdf]

Supplementary materials

Figures :3

Tables : 0

Supplementary Figure Legends :

Supplementary Figure 1: Risk of bias traffic plot

Supplementary Figure 2: Risk of bias summary plot

Supplementary Figure 3: Funnel plot

Systematic literature search algorithm :

Scopus : ("myasthenia" OR "myasthenia gravis" OR "generalized myasthenia") AND "diabetes mellitus") OR (“cardiovascular”) or” hyperglycemia”

PubMed : (((myasthenia) OR (myasthenia gravis) OR (generalized myasthenia)) AND ((diabetes mellitus) OR "cardiovascular" or hyperglycemia))

Supplementary Figure 1

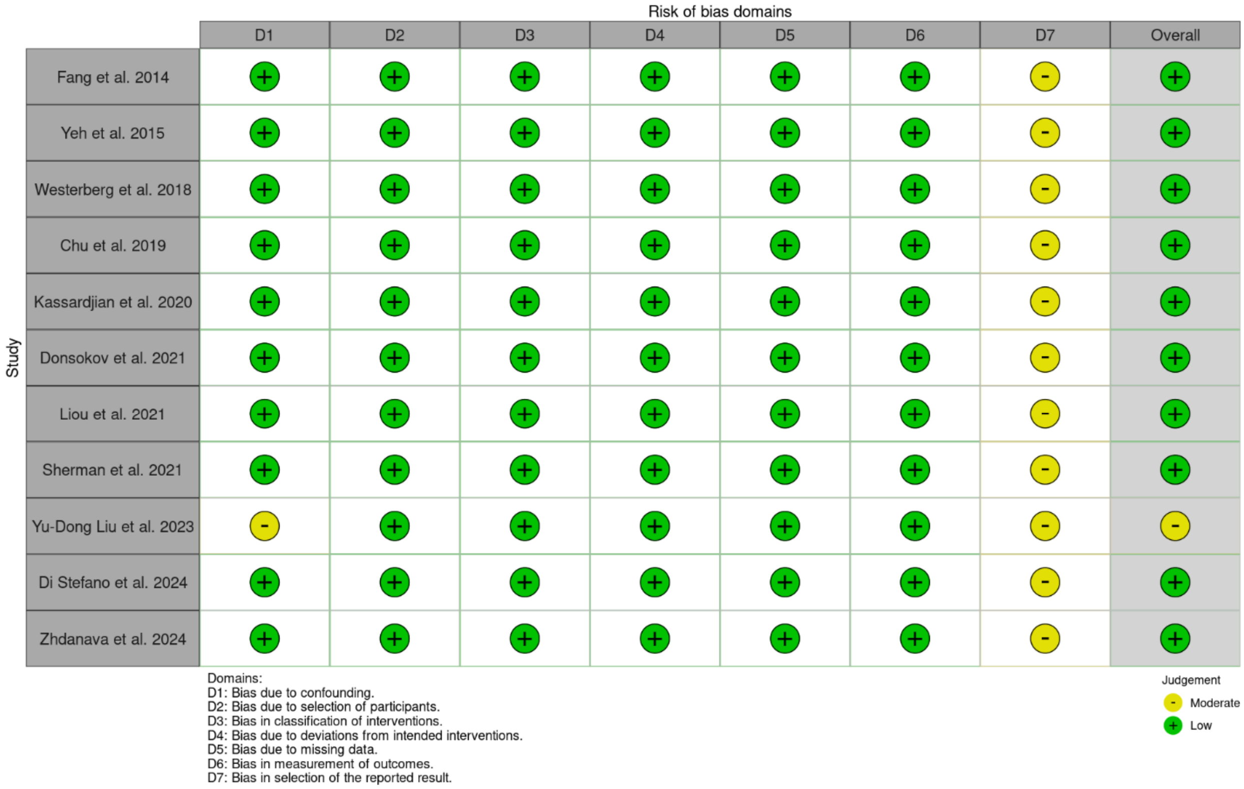

Supplementary Figure S2

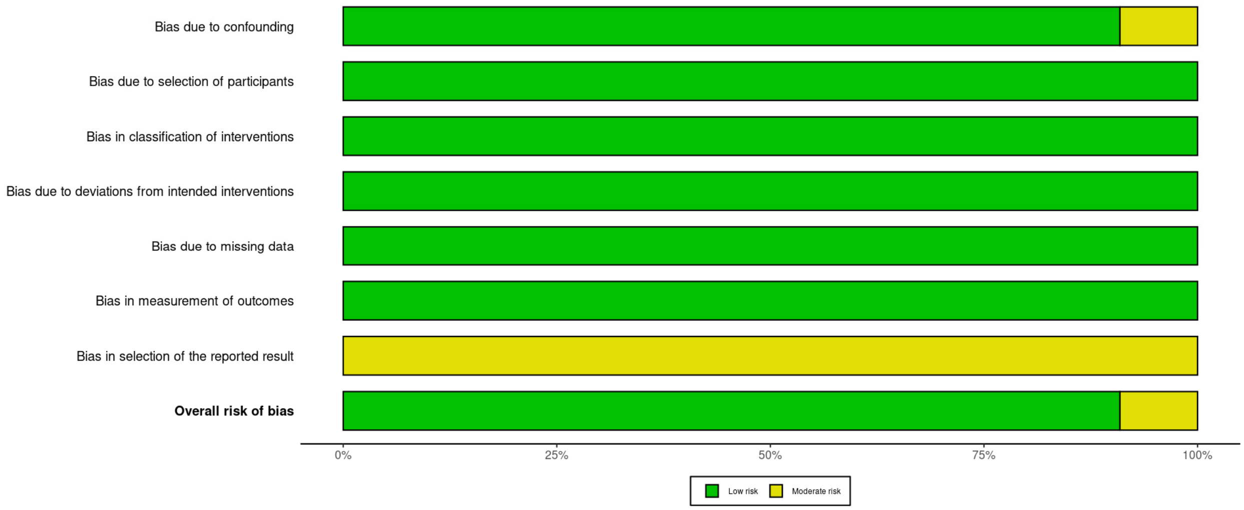

Supplementary Figure S3

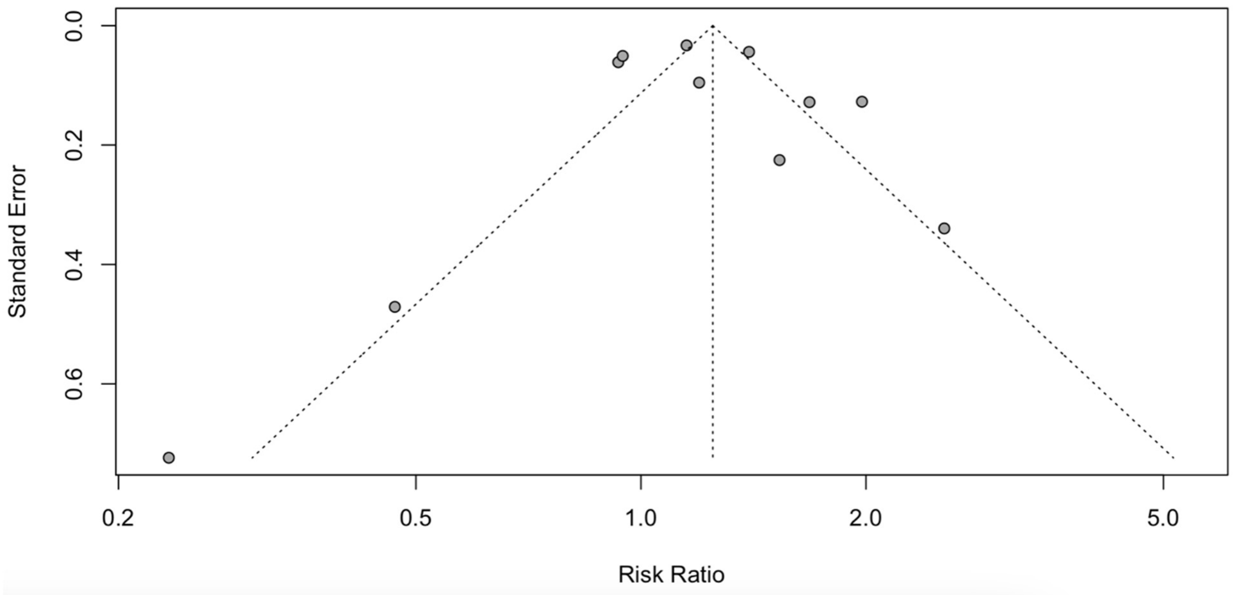
Standard Error
0.0
0.2
0.4
0.6
0.2
0.5
1.0
2.0
5.0
Risk Ratio
